# Hidden Burden of Fallopian Tube Endometriosis: Prevalence and Associations with Pelvic Pathology

**DOI:** 10.3390/jcm15031136

**Published:** 2026-02-01

**Authors:** Farr Nezhat, Pegah Rashidian, Shadi Seraji, Esra Demirel, Shahidul Islam, Poonam Khullar, Camran Nezhat

**Affiliations:** 1 Nezhat Surgery for Gynecology/Oncology, New York, NY 10128, USA; 2 Department of OB/GYN, NYU Grossman Long Island School of Medicine, Mineola, NY 11501, USA; 3 Weill Cornell Medical College, Cornell University, New York, NY 10021, USA; 4 Vali-e-Asr Reproductive Health Research Center, Family Health Research Institute, Tehran University of Medical Sciences, Tehran 1419733141, Iran; pegah.rashidian@rocketmail.com; 5 Department of Obstetrics and Gynecology, NYU Grossman Long Island School of Medicine, Mineola, NY 11501, USA; shadi.seraji@nyulangone.org (S.S.); esra.demirel@nyulangone.org (E.D.); 6 Northwell Health, New Hyde Park, NY 11040, USA; sislam22@northwell.edu; 7 Department of Pathology, NYU Grossman Long Island School of Medicine, Mineola, NY 11501, USA; poonam.khullar@nyulangone.org; 8 Stanford University Medical Center, Palo Alto, CA 94305, USA; camran@camrannezhatinstitute.com; 9 University of California San Francisco, San Francisco, CA 94143, USA; 10 Camran Nezhat Institute, Center for Special Minimally Invasive and Robotic Surgery, Woodside, CA 94061, USA

**Keywords:** endometriosis, fallopian tube, fertility, tubo-ovarian cancer, hydrosalpinx

## Abstract

**Background/Objectives:** Fallopian tube endometriosis is frequently underreported due to its nonspecific presentation, limitations in imaging detection, and the fact that histologic diagnosis often requires the submission of the entire fallopian tube for microscopic analysis, which is not routinely performed. This study investigates the prevalence of fallopian tube endometriosis on histologic evaluation in patients undergoing laparoscopic salpingectomy for benign gynecologic conditions, and evaluates its association with intraoperative findings such as endometriomas, hydrosalpinx, and peri-tubal adhesions. **Methods:** A prospective cohort study was conducted from September 2021 to December 2024 at NYU Grossman Long Island School of Medicine, including 80 women ages 18–65 years undergoing unilateral or bilateral salpingectomy. Fallopian tubes were entirely submitted for histologic examination and were evaluated in cross-sections for presence of endometriosis. The association between the presence of fallopian tube endometriosis, the stage of endometriosis, the presence of endometriomas, hydrosalpinx, and tubal adhesions was analyzed, with significance defined as *p* < 0.05. **Results:** 47 women were found to have biopsy-proven pelvic endometriosis (58.75%). The prevalence of fallopian tube endometriosis was 42.50% (34/80) in the total study population and 72.34% (34/47) among patients with pelvic endometriosis. The most commonly involved layer was the serosa (75.5%), followed by the muscularis (46.9%) and mucosa (18.4%). Fallopian tube endometriosis was significantly associated with endometriomas (*p* < 0.0001), hydrosalpinx (*p* < 0.0001), and tubal adhesions (*p* < 0.0001). It was also strongly correlated with disease severity, with 92.9% of patients with stage 4 endometriosis exhibiting tubal involvement. **Conclusions:** Fallopian tube endometriosis is more prevalent than previously recognized and shows a strong association with advanced-stage endometriosis, endometriomas, hydrosalpinx, and tubal adhesions.

## 1. Introduction

Endometriosis is a chronic, inflammatory disease characterized by the presence of endometrial-like tissue outside the endometrial cavity, seen in approximately 10% of reproductive-aged women, although some estimates suggest that the true prevalence may be even higher [1,2]. Endometriosis has been associated with a range of reproductive sequelae, including infertility and adverse pregnancy outcomes [3]. While endometriosis predominantly affects the ovaries and other deep pelvic structures, its manifestation within the fallopian tubes remains largely underreported [4,5]. Tubal endometriosis is classified into three distinct histopathological subtypes. The most common subtype involves endometrial implants infiltrating into the serosa or subserosa of the fallopian tube, often coexisting with other pelvic endometriosis and considered a type of peritoneal endometriosis. This invasion can lead to fibrosis and potential tubal occlusion, resulting in hydrosalpinx and ectopic pregnancy [5]. The second subtype, known as endometrial colonization, occurs when endometrial tissue infiltrates the tubal mucosa, resulting in intraluminal endometriosis [5]. The third subtype, post-salpingectomy endometriosis, develops in the proximal residual segment of the fallopian tube following tubal ligation or salpingectomy, typically manifesting one to four years post-surgery [5].

The reported incidence of fallopian tube endometriosis varies significantly, depending on the diagnostic approach. Its prevalence has been estimated to be 3.84–11.82% by intraoperative visual assessment [6,7,8]. However, recent studies based on histological evaluation suggest a substantially higher incidence, ranging from 37.37% to 42.5% [9,10]. This discrepancy underscores the limitations of macroscopic surveys and highlights the need for systematic histopathological evaluation to more accurately determine the true burden of tubal endometriosis [9,11].

In our previous retrospective study, we reported that microscopic fallopian tube endometriosis occurs at a significantly higher rate than macroscopic visible disease. While this study evaluated endometriosis within fallopian tubes microscopically, the study did not characterize the distribution of endometriosis within the fallopian tubes [9]. Our current study aims to determine the prevalence of endometriosis in different layers and segments of the fallopian tube through systematic serial histopathological examination in patients undergoing laparoscopic unilateral or bilateral salpingectomy for benign gynecologic conditions, with a secondary objective of assessing its association with intraoperative and pathological findings. Characterizing this distribution and its correlation with intraoperative findings such as hydrosalpinx, endometriomas, and peritubal adhesions may help predict microscopic involvement, guide surgical decision-making, and elucidate implications for fertility and cancer risk [9].

## 2. Materials and Methods

### 2.1. Design and Participants

A prospective cohort study was conducted from September 2021 to December 2024 at the NYU Grossman Long Island School of Medicine to investigate the incidence and histological distribution of endometriosis within fallopian tubes. The study includes women ages 18 to 65 years who underwent laparoscopic unilateral or bilateral salpingectomy for benign gynecologic indications. The procedures were performed in four main clinical scenarios: (1) opportunistic salpingectomy at time of hysterectomy for benign gynecologic conditions including pelvic pain secondary to endometriosis, abnormal uterine bleeding, leiomyomata, and adenomyosis; (2) the management of adnexal masses; (3) the treatment of tubal damage or hydrosalpinx in patients undergoing infertility evaluation or in vitro fertilization; and (4) elective sterilization. Patients could meet more than one of these indications. Women with a history of malignancy or prior bilateral salpingectomy were excluded. The study was approved by the institutional review board (IRB), and all participants provided informed consent prior to data collection.

This article is a revised and expanded version of a paper entitled “Incidence of Fallopian Tube Endometriosis and Its Association with Endometriomas, Hydrosalpinx, and Peritubal Adhesions: A Prospective Cohort Study,” which was presented at the 2024 Annual Meeting of the American Society for Reproductive Medicine (ASRM), and published in abstract form in Fertility and Sterility (October 2024) [12].

### 2.2. Data Collection

Data was collected on preoperative baseline clinical characteristics such as age, body mass index, reproductive history, history of infertility, and prior pelvic surgery. Additionally, indications for the current gynecological surgery were recorded, including pelvic pain, abnormal uterine bleeding, leiomyomata, adenomyosis, endometriosis, adnexal mass, infertility, hydrosalpinx, and sterilization. Surgeries were performed by two experienced surgeons (F. N. and S. S.), both of whom have extensive experience in minimally invasive surgery and treatment of endometriosis. Detailed operative findings were recorded, including the presence of fallopian tube endometriosis and various clinical factors such as the stage of endometriosis, presence of endometriomas, hydrosalpinx, and tubal adhesions. The staging of endometriosis was performed according to the Revised American Society for Reproductive Medicine staging system [13], based on intraoperative visual inspection of lesion location, size, and severity, with pathological confirmation provided by histopathologic evaluation. Following salpingectomy, the entire fallopian tubes were submitted for histological examination to assess their involvement. Serial cross-sections were obtained and analyzed by a single pathologist (P.K.) for consistency. The fallopian tubes were examined in four segments: (A) isthmus (proximal), (B) ampulla (mid-segment), (C) infundibulum (distal), and (D) fimbriae. Each segment was assessed for endometriosis involvement across all layers of the fallopian tube including serosa, muscularis, and mucosa. Histological assessment was performed using hematoxylin and eosin (H&E) staining only; immunohistochemical or other special stains were not performed.

### 2.3. Statistical Analysis

Patient demographics, surgical findings, and histopathological results were systematically recorded and analyzed. Descriptive statistics were computed for each group, with continuous variables summarized using the mean ± standard deviation and categorical variables presented as frequencies and percentages. The Chi-square or Fisher’s exact test were used to compare categorical variables, as appropriate, while continuous variables were compared between groups using the independent two-sample *t*-test. Statistical significance was set at a *p*-value of less than 0.05. All analyses were performed using SAS software, version 9.4 (SAS Institute Inc., Cary, NC, USA).

## 3. Results

A total of 80 patients were included in the study, with no withdrawals or losses to follow-up. All participants underwent salpingectomy: 72 underwent bilateral salpingectomy, 3 underwent left salpingectomy, and 5 underwent right salpingectomy, resulting in a total of 152 fallopian tubes for histopathological evaluation.

Among the 80 patients included in the study, histopathological evaluation confirmed pelvic endometriosis in 47 patients (58.8%). Histological examination revealed fallopian tube endometriosis in 34 patients, 42.5% of the total cohort and in 72.3% of those with biopsy-proven pelvic endometriosis. Four patients (5%) exhibited isolated fallopian tube endometriosis without evidence of pelvic involvement. Of the 152 fallopian tubes examined, only 11 (7.2%) showed macroscopic evidence of endometriosis, and 49 (32.2%) demonstrated histologically confirmed endometriotic lesions. Demographic and clinical characteristics of the study population are summarized in Table 1.

Given that the study population included patients with a range of gynecologic diagnoses—some of which may confer a higher likelihood of fallopian tube endometriosis—additional descriptive analysis was conducted to compare the macroscopic and histological prevalence across these preexisting diagnoses. The findings are presented in Table 2.

**Figure 1:**
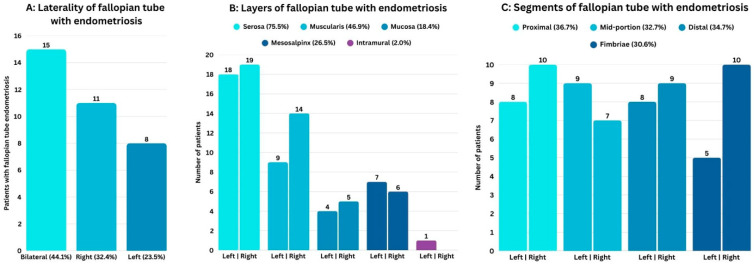
Distribution of fallopian tube endometriosis according to (**A**) laterality, (**B**) anatomical layers involved, and (**C**) affected tubal segments.

Among the 49 fallopian tubes positive for endometriosis, the most frequently affected layer was the serosa (75.5%), followed by the muscularis (46.9%), and mucosa (18.4%), with multiple involved sites in some tubes. Transmural endometriosis was observed in one tube. With the exception of two cases, all instances of muscularis involvement were accompanied by concurrent serosal involvement. Along the length of the involved fallopian tubes, endometriosis was found in the proximal (isthmic) segment in 36.7%, mid-portion (ampulla) in 32.7%, distal segment (infundibulum) in 34.7%, and the fimbriated end in 30.6%. Data on the laterality, layers, and segments of fallopian tube endometriosis are provided in Figure 1.

In our study, 37 (46.3%) patients were found to have fallopian tube adhesions, with 25 patients presenting with bilateral adhesions, 7 with left-sided peritubal adhesions, and 5 with right-sided peritubal adhesions. Additionally, 23 (28.8%) patients were found to have hydrosalpinx, of whom 16 had bilateral involvement, 4 had right-sided hydrosalpinx, and 3 had left-sided hydrosalpinx. Both intraoperative findings—tubal adhesions and hydrosalpinx—were significantly associated with fallopian tube endometriosis on final pathology (*p* < 0.0001). Furthermore, 27 (33.8%) of the patients were found to have ovarian endometriomas, with 11 presenting with bilateral endometriomas, 7 with left-sided involvement, and 9 with right-sided involvement. The laterality of endometriomas was significantly associated with the laterality of fallopian tube endometriosis (left: *p* = 0.0002, right: *p* < 0.0001). The associations between fallopian tube endometriosis and the presence of endometriomas, hydrosalpinx, and tubal adhesions are summarized in Table 3.

Among the patients with bilateral fallopian tube endometriosis (n = 15), all were diagnosed with stage 4 endometriosis. The presence of fallopian tube endometriosis showed a significant correlation with advanced disease. Fallopian tube endometriosis was identified in 92.9% of patients with stage 4 endometriosis, compared to 40.0% in stage 3, 33.3% in stage 2, and 45.5% in stage 1 (*p* < 0.0001). Notably, four patients had endometriosis confined exclusively to the fallopian tubes, all of whom were classified as having stage 1 disease. The clinical indications and anatomical locations of fallopian tube involvement in these cases were as follows: (1) mesosalpinx involvement of left tubal remnant, in a postmenopausal patient with a left ovarian cyst and a history of right salpingo-oophorectomy and left salpingectomy; (2) mucosal involvement of the right tube in a patient undergoing surgery for infertility and hydrosalpinx; (3) mesosalpinx involvement of the right tube in a patient with fibroids, abnormal uterine bleeding, polyps, and suspected endometriosis undergoing hysterectomy and salpingectomy; and (4) serosal involvement of the right tube in a patient with a right dermoid cyst who underwent right salpingo-oophorectomy. Details on the presence and laterality of fallopian tube endometriosis by postoperative endometriosis stage are summarized in Table 4.

## 4. Discussion

In this study, histologically proven fallopian tube endometriosis was present in 42.5% of patients undergoing salpingectomy and in 72.34% of those with pelvic endometriosis. The most frequently affected histological layer was the serosa (75.5%), followed by the muscularis (46.9%) and the mucosa (18.4%). Endometriotic lesions were distributed relatively uniformly across the proximal (36.7%), mid-portion (32.7%), distal (34.7%), and fimbriated (30.6%) segments of the fallopian tube. Despite clinical impressions, endometriosis of the fimbriated end was not uncommon in this series. In two fallopian tubes, muscularis involvement was observed without concurrent serosal involvement, a pattern potentially analogous to uterine adenomyosis and possibly representing a form of fallopian tube adenomyosis. Interestingly, in four patients, the fallopian tube was the only site of pelvic endometriosis, including one case with isolated mucosal involvement. Additionally, associations were observed between fallopian tube endometriosis and endometriomas, tubal adhesions, hydrosalpinx, and advanced-stage endometriosis.

In our study, patients with fallopian tube endometriosis were younger than those without fallopian tube endometriosis, which may reflect the higher prevalence of active or clinically apparent endometriosis in reproductive-aged women, as reported in prior studies [10]. However, the consistent association of fallopian tube endometriosis with advanced-stage disease and related anatomic findings suggests that these relationships are driven primarily by disease extent rather than age alone. In addition, adenomyosis appeared less frequent among patients with fallopian tube endometriosis compared to those without, though the difference was not statistically significant. This finding may reflect variability in patient selection, surgical indications, or the independent pathophysiology of adenomyosis and fallopian tube endometriosis. Larger studies are needed to determine whether this represents a true clinical trend or a chance observation.

The findings of the study are consistent with recent literature that underscores the underestimated prevalence of fallopian tube endometriosis when relying solely on macroscopic visual diagnosis. McGuinness et al. (2020) reported that the incidence of tubal endometriosis among patients with endometriosis was 11–12% macroscopically, but 42.5% microscopically [9]. Additionally, Qi et al. (2019) underscored the importance of histopathological confirmation for an accurate diagnosis, suggesting that systematic, complete tubal examination should become standard practice for patients undergoing gynecologic surgery [10]. In contrast to our findings where the serosa (75.5%) was the most frequently affected layer, Qi et al. reported a predominance of mucosal involvement (54.7%) in their overall cohort, with lower rates of serosal involvement (32.3%) and muscularis (myosalpinx) involvement (6.2%) [10]. Additionally, combined mucosal and serosal lesions were observed in 6.8% of cases. These differences may reflect variations in study populations, surgical indications, or disease severity.

Regarding laterality, previous studies have reported conflicting results, with some indicating a higher prevalence on the left side and others showing a right-sided predominance [7,14]. Although our study observed a right-sided predominance, it is likely that variations in patient numbers and factors as well as diagnostic methods between studies contribute to discrepancies in the reported laterality of tubal endometriosis.

The associations between fallopian tube endometriosis and endometriomas, tubal adhesions, hydrosalpinx, and advanced-stage endometriosis observed in our study are supported by previous research. A cross-sectional study reported that patients with fallopian tube endometriosis had a significantly increased risk of hydrosalpinx or hematosalpinx, with a prevalence of 43% [10]. This is likely due to the scarring and obstruction of the fallopian tubes by endometriosis, causing the accumulation of fluid or blood in the fallopian tubes [10]. In 2024, Seraji et al. demonstrated that the presence of ovarian endometriomas was associated with a higher stage of disease, with stage IV endometriosis being the most commonly observed stage in these patients [15] Similarly, our findings show that tubal endometriosis is highly associated with stage IV disease.

Endometriosis has a well-established association with infertility, with approximately 30–50% of affected women experiencing infertility [16]. Furthermore, the negative impact of endometriomas, and tubal pathology, such as hydrosalpinx, on fertility has been well established in previous studies [17,18,19]. Surgical resection or ablation of endometriotic lesions has been consistently shown to improve fecundity in infertile women, even among those with prior failed in vitro fertilization attempts [19,20,21]. Furthermore, in patients with endometriosis and associated peritubal adhesions or hydrosalpinx, laparoscopic correction has been associated with improved clinical pregnancy and live birth rates following assisted reproductive technologies [22]. Based on the associations identified in our study—particularly between fallopian tube endometriosis and the presence of endometriomas, hydrosalpinx, and tubal adhesions—we propose that the potential role of salpingectomy in selected women with unexplained infertility warrants further investigation. Given the observational nature of this study, these considerations remain hypothesis-generating and should not be interpreted as clinical recommendations. Importantly, the management of microscopic endometriosis may need to be tailored according to anatomical location and functional significance. In the bowel, invisible microscopic endometriosis implants may extend several centimeters beyond visible lesions, making complete microscopic excision impractical and supporting a surgical strategy focused on removal of macroscopic disease only, as demonstrated in prior histopathological studies [23]. In contrast, the fallopian tube represents a discrete, functionally critical structure whose removal is technically feasible and already routinely performed in selected clinical contexts, such as hydrosalpinx prior to assisted reproduction or opportunistic salpingectomy for cancer risk reduction [22,24]. In this setting, the presence of histologically confirmed tubal endometriosis—particularly when associated with tubal dysfunction—raises the question of whether salpingectomy could offer therapeutic benefit in carefully selected patients. Prospective studies and randomized clinical trials are needed to clarify the reproductive and long-term outcomes of this approach.

Another finding of our study was the identification of isolated fallopian tube endometriosis in four patients, in whom no other pelvic endometriotic lesions were detected, suggesting that fallopian tube endometriosis may occur as a primary or early manifestation of the disease rather than solely as an extension of advanced pelvic endometriosis. Although the small number of cases precludes definitive conclusions regarding clinical impact, isolated tubal involvement may have important implications for symptomatology and fertility. Subtle tubal pathology may contribute to tubal dysfunction through impaired ciliary activity, altered tubal peristalsis, local inflammation, or luminal distortion, even in the absence of overt pelvic disease. This may partly explain cases of unexplained infertility or persistent symptoms despite otherwise normal laparoscopic findings [25]. The identification of isolated fallopian tube endometriosis further underscores the limitations of macroscopic inspection alone and highlights the importance of systematic histopathological evaluation of the fallopian tubes.

Moreover, women with endometriosis have an increased likelihood of developing ovarian cancer, with a particularly elevated risk for endometrioid and clear cell ovarian carcinoma [11,26,27,28]. Current evidence also suggests that fallopian tubes play a critical role in the development of type II ovarian cancer [29,30,31]. Currently, opportunistic salpingectomy is recommended to reduce the risk of high-grade serous ovarian carcinoma [11,32]. Although this approach is not yet established for preventing endometriosis-associated malignancies, reports of extra-ovarian malignant transformation of endometriosis at several sites—including the cul-de-sac [33], vagina [34], bowel [35], and ureter [36] —as well as isolated cases of malignant transformation arising from tubal endometriosis [37,38,39], suggest the need to investigate appropriate preventive strategies targeting the fallopian tube. Evidence shows that tubal ligation and hysterectomy are associated with decreased ovarian cancer risk, the effect being somewhat stronger for non-serous tumors, with greater reductions in the incidence of endometrioid (OR 0.48, 95% CI: 0.40–0.59) and clear cell carcinoma (OR 0.52, 95% CI: 0.40–0.67) compared to high-grade serous carcinoma (OR 0.80, 95% CI: 0.73–0.89). This is likely due to interruption of retrograde menstruation and inflammatory signaling from the lower genital tract [40]. Moreover, large prospective cohort data indicate that both tubal ligation and hysterectomy are associated with decreased ovarian cancer risk, with the effect being somewhat stronger for non-serous tumors [41]. However, these studies did not specifically evaluate the role of salpingectomy in risk reduction.

A population-based study of 78,893 women with endometriosis demonstrated that those with ovarian endometriomas or deep infiltrating endometriosis had a 9.7-fold increase overall risk of ovarian cancer, including a 19-fold increase for type I and a 3.7-fold increase for type II ovarian cancer [42].

In our study, although no cases of malignancy were included, we observed a higher-than-expected prevalence of fallopian tube endometriosis. These lesions were often associated with endometriomas and showed deep invasion beyond the serosa into the muscularis and mucosa, indicating a more extensive and possibly more aggressive disease pattern. This deep muscularis involvement, reminiscent of uterine adenomyosis, may represent a more invasive form of tubal endometriosis with potential for aggressive behavior. Taken together with prior research, our findings highlight the current lack of clinical trials and underscore the need to evaluate whether removal of fallopian tubes affected by endometriosis could reduce the risk of malignant transformation.

Future longitudinal studies involving larger cohorts are crucial to assess the long-term effects of surgical interventions, such as bilateral salpingectomy on clinical outcomes. These studies should aim to determine whether such procedures may play a role in reducing the risk of tubo-ovarian cancer and mitigating the adverse impact of fallopian tube endometriosis on fertility or assisted reproductive technologies. Advances in artificial intelligence and emerging technologies may further support these research efforts [43].

This study has several notable strengths. We investigated fallopian tube endometriosis across multiple segments (proximal, mid, distal, fimbriae) and histological layers (serosa, muscularis, mucosa), providing a detailed characterization of disease distribution. All surgeries were performed by surgeons with extensive expertise in minimally invasive surgery and endometriosis treatment, ensuring a high standard of surgical technique and consistency in procedural execution. Additionally, the prospective study design and rigorous histopathological examination allowed for precise evaluation of fallopian tube involvement. Furthermore, the associations observed between fallopian tube endometriosis and other intraoperative findings provide valuable insights into its pathophysiology and clinical significance.

Limitations include the single-center design, which may restrict the generalizability of the findings to broader populations. The relatively small sample size also limits statistical power and may not fully capture the spectrum of fallopian tube endometriosis across different patient populations. Furthermore, the cohort was heterogeneous, comprising patients with diverse surgical indications (e.g., abnormal uterine bleeding, infertility, chronic pain) and a wide age range. Because all patients who underwent salpingectomy had underlying pelvic pathology, the observed prevalence may not be representative of the general population. Another important limitation is the lack of long-term follow-up data, particularly regarding fertility outcomes, disease progression, and potential malignant transformation. Additionally, molecular and immunohistochemical analyses were not performed. The absence of biomarker evaluation limits insight into the molecular pathways, hormonal responsiveness, and the potential malignant transformation of fallopian tube endometriosis.

## 5. Conclusions

In conclusion, this study confirms the previously reported high prevalence of fallopian tube endometriosis and underscores the importance of histological evaluation of fallopian tubes for endometriosis in patients undergoing gynecologic surgery; relying solely on intraoperative visualization may lead to significant underdiagnosis. Fallopian tube endometriosis is often associated with advanced-stage disease, endometriomas, hydrosalpinx, and tubal adhesions. These findings underscore the clinical significance of fallopian tube endometriosis, particularly regarding its impact on reproductive outcomes, disease progression, and potential oncologic risks. Future longitudinal studies with larger cohorts are necessary to assess the long-term effects of salpingectomy on clinical outcomes such as tubo-ovarian cancer risk and reproductive outcomes. Advances in artificial intelligence and emerging technologies hold promise for achieving these goals and enhancing the precision of treatments.

## Figures and Tables

**Table 1:** Demographic and clinical characteristics of the study population.

		Fallopian Tube Endometriosis (n = 34)	No Fallopian Tube Endometriosis (n = 46)	*p*-Value
Age; y		39.90 ± 6.40	46.0 ±8.75	0.001
BMI; kg/m^2^		29.28 ± 4.91	29.48 ±6.51	0.681
Reproductive history	Gravidity 0	14 (41.2)	8 (17.4)	0.018
	Gravidity 1–2	13 (38.2)	16 (34.8)	
	Gravidity ≥ 3	7 (20.6)	22 (47.8)	
	Parity 0	18 (52.9)	14 (30.4)	0.045
	Parity 1–2	13 (38.2)	19 (41.3)	
	Parity ≥ 3	3 (8.8)	13 (28.3)	
History of pelvic surgery		23	28	0.533
History of hormone therapy	None	10	29	0.012
	Combined contraceptive pills	12	11	
	Progestin-only pills	8	5	
	GnRH-agonists	4	3	
	GnRH-antagonists	6	1	
Indications for surgery *	Pelvic pain	28	28	0.068
	AUB	23	25	0.230
	Leiomyomata	16	33	0.025
	Adenomyosis	7	15	0.234
	Endometriosis	22	12	0.00055
	Adnexal mass	3	7	0.393
	Infertility	20	5	<0.00001
	Hydrosalpinx	8	2	0.010
	Sterilization	0	4	

All the data in this table are expressed as mean ± standard deviation or numbers and percentages (Percentages may not sum to 100% due to rounding). * Some patients may have had multiple indications for surgery. AUB: Abnormal Uterine Bleeding; BMI: Body Mass Index; GnRH: Gonadotropin-Releasing Hormone; n: Number; y: Year.

**Table 2:** Macroscopic and histological prevalence of fallopian tube endometriosis in each preexisting diagnosis.

Diagnosis *	Number of Patients in Each Group (n)	Macroscopic FTE, n (%)	Histological FTE, n (%)
Total population	80	6 (7.5)	34 (42.5)
Pelvic pain	56	6 (10.7)	28 (50.0)
AUB	48	5 (10.4)	23 (47.9)
Leiomyomata	49	2 (4.1)	16 (32.7)
Adenomyosis	22	3 (13.6)	7 (31.8)
Endometriosis	34	6 (17.6)	22 (64.7)
Adnexal mass	10	0 (0.0)	3 (30.0)
Infertility	25	4 (16.0)	20 (80.0)
Hydrosalpinx	10	1 (10.0)	8 (80.0)
Sterilization	4	0 (0.0)	0 (0.0)

AUB: Abnormal Uterine Bleeding; FTE: Fallopian Tube Endometriosis; n: Number; %: Percent. All the data in this table are presented as numbers and percentages. * Some patients may have had multiple surgical indications or concurrent preoperative diagnoses.

**Table 3:** Association of fallopian tube endometriosis with ovarian endometriomas, hydrosalpinx, and tubal adhesions.

Factor	Presence of Fallopian Tube Endometriosis *		Fisher’s Exact Test; Two-Sided *p*-Value
	Yes, n (%)	No, n (%)	
Presence of Fallopian Tube Adhesions	29 (85.29)	8 (17.78)	<0.0001
Left Hydrosalpinx	12 (52.17)	7 (12.28)	0.0003
Right Hydrosalpinx	17 (65.38)	3 (5.56)	<0.0001
Presence of Fallopian Tube Endometrioma	26 (76.47)	1 (2.17)	<0.0001
Left Fallopian Tube Endometrioma	12 (52.17)	6 (10.53)	0.0002
Right Fallopian Tube Endometrioma	17 (65.38)	3 (5.56)	<0.0001

n: Number; %: Percent. * The presence of fallopian tube endometriosis is defined based on the laterality of the associated factor being investigated, resulting in a sample size of 34 for bilateral, 23 for left-sided, and 26 for right-sided findings.

**Table 4:** Distribution and laterality of fallopian tube endometriosis by postoperative endometriosis stage.

Fallopian Tube Endometriosis by Postop Stage of Endometriosis						
Laterality of Fallopian Tube Endometriosis	Postop Stage of Endometriosis					
	N/A, n (%)	Stage 1, n (%)	Stage 2, n (%)	Stage 3, n (%)	Stage 4, n (%)	Total
None	33 (71.74)	6 (13.04)	2 (4.35)	3 (6.52)	2 (4.35)	46
Left	0 (0.00)	2 (25.00)	0 (0.00)	2 (25.00)	4 (50.00)	8
Right	0 (0.00)	3 (27.27)	1 (9.09)	0 (0.00)	7 (63.64)	11
Bilateral	0 (0.00)	0 (0.00)	0 (0.00)	0 (0.00)	15 (100.00)	15
Total	33 (41.25)	11 (13.75)	3 (3.75)	5 (6.25)	28 (35.00)	80

n: Number; N/A: Not Applicable; %: Percent.

## Data Availability

The data presented in this study are available on request from the corresponding author due to restrictions imposed by Institutional Review Board approval and the need to protect patient confidentiality.

## Abbreviations

ART	Assisted Reproductive Technologies
ASRM	American Society for Reproductive Medicine
IRB	Institutional Review Board
H&E	Hematoxylin and Eosin
